# Impact of Occupational and Extra-Professional Exposure Across the Different Waves of the Pandemic on the Risk of SARS-CoV-2 Infection Among Healthcare Workers—The ORCHESTRA Project

**DOI:** 10.3390/healthcare14131872

**Published:** 2026-06-26

**Authors:** Gianluca Spiteri, Lorena Torroni, Angela Contri, Angela Carta, Filippo Liviero, Anna Volpin, Maria Luisa Scapellato, Luca Cegolon, Francesca Rui, Marcella Mauro, Paola Ferri, Fabriziomaria Gobba, Giuseppe Verlato, Stefano Porru, Alberto Modenese

**Affiliations:** 1 Occupational Medicine Unit, University Hospital of Verona, 37134 Verona, Italy; gianluca.spiteri@aovr.veneto.it (G.S.);; 2 Departmental Faculty of Medicine, Saint Camillus International University of Health and Medical Sciences, 00131 Rome, Italy; 3 Department of Biomedical, Metabolic and Neural Sciences, University of Modena and Reggio Emilia, 41125 Modena, Italy; angela.contri@unimore.it (A.C.); alberto.modenese@unimore.it (A.M.); 4 Section of Occupational Medicine, Department of Diagnostics and Public Health, University of Verona, 37134 Verona, Italy; 5 Occupational Medicine Unit, University Hospital of Padova, Via Giustiniani 2, 35128 Padova, Italy; 6 Department of Cardiac Thoracic Vascular Sciences and Public Health, University of Padova, 35128 Padua, Italy; 7 Unit of Occupational Medicine, Department of Medical Science, University of Trieste, 34149 Trieste, Italy; luca.cegolon@units.it (L.C.);; 8 Unit of Epidemiology and Medical Statistics, Department of Diagnostics and Public Health, University of Verona, 37134 Verona, Italy; giuseppe.verlato@univr.it

**Keywords:** healthcare workers, SARS-CoV-2, COVID-19, extraprofessional exposure, pandemic

## Abstract

**Background/Objectives**: Healthcare workers (HCWs) were the most exposed job category to SARS-CoV-2, due to patient care, HCW-to-HCW transmission, and community exposure. However, the relative relevance of each source is still debated. To address this issue, this study investigated the dynamics of the professional and extra-professional determinants of infection across the pandemic among a large, multicenter cohort of HCWs. **Methods**: The study included 5576 HCWs from four Italian University Hospitals within a European Project, called ORCHESTRA. Socio-demographic and clinical data were collected retrospectively via online surveys from March 2020 to September 2022. Factors associated with SARS-CoV-2 infection during different pandemic periods were evaluated by a multinomial logistic regression model. Results were expressed as Relative Risk Ratios (RRR). **Results**: The cumulative incidence was 46.2%. The highest incidence period was the Omicron phase (OVP) (69.7%). The extra-professional source was the most reported (34.3%), followed by the occupational (26.8%). However, in almost 40%, the source was undetected. The RRR for occupational exposures was 0.39 (95% CI 0.25–0.61) during the Pre-Omicron variant Period (POP) and even lower (0.22, 95% CI 0.16–0.29) in the OVP, as compared to extra-professional exposures, using the Pre-Vaccination Period (PVP) as reference. **Conclusions**: The dominant source of infection among HCWs changed over time. While occupational contacts were more frequent during PVP, it significantly waned over the subsequent pandemic phases. Implementing procedures and guidelines to prevent infection, even outside the workplace during pandemics, would reduce the spread of infection among HCWs and patients.

## 1. Introduction

Healthcare workers (HCWs) paid a very high toll in terms of morbidity and mortality during the SARS-CoV-2 pandemic. The World Health Organization has estimated that between 80,000 and 180,000 healthcare workers died from COVID-19 between January 2020 and May 2021 alone [1]. Matz et al. assessed excess mortality by occupational group in England and Wales during 2020. They reported the highest excess mortality among HCWs (13.3%) [2]. Moreover, as reported by Ferland et al. in an analysis of COVID-19 cases in 9 European Countries, at least in the pre-vaccination period (PVP), HCWs were also more prone to infection (Adjusted Attack Ratios in HCWs–AAR = 3.0, 95% CI 1.1–3.2), hospitalization (AAR = 1.8, 95% CI 1.2–2.7), and ICU admission (AAR = 1.0, 95% CI 1.1–3.2), as compared to general population [3]. The fact that HCWs were the most exposed job category to SARS-CoV-2 was also confirmed by Laskaris et al. The Authors investigated the source of infection among 1839 workers across different sectors who tested positive during March-November 2020. The percentage of employees in nursing and residential care facilities was more than double the average for all sectors (65.2% vs. 30.4%) [4]. As expected, these effects were directly related to the tasks of direct patient care performed by HCWs [5]. In agreement with this hypothesis, in a previous study conducted among HCWs involved in the ORCHESTRA Project, we found that HCWs providing direct patient care, such as nurses, were at higher risk of infection during the PVP than administrative staff [6]. However, its impact seemed to wane during subsequent waves, especially after vaccination, suggesting that the determinants of risk changed over time. Although several studies have investigated other factors that influence infection risk, the role of extraprofessional exposures among HCWs remains unclear. Indeed, Wight et al. reported that occupational exposure was associated with infection only in 3.55% of the 3694 HCWs employed at Mayo Clinic (USA) who tested positive between July and December 2020. On the other hand, household and community exposures accounted for more the 42% of the cases (27.1% and 15.6%, respectively), representing the most relevant routes of exposure in that population [7]. Shaw et al. found similar results in a Cohort of 8766 American HCWs, with more than 4400 cases from March 2020 to May 2022. Indeed, household and community exposures were dominant (26.8% and 10.3%), while occupational exposures were confirmed as residual (<5%) [8]. On the contrary, in a very large HCWs population (n = 462,453), Billock et al. identified occupational exposure as the main cause of infection (52%), and its influence was greater at the highest community levels. In particular, the adjusted Prevalence Ratio (aPR) for reporting occupational exposure before infection was 1.31 (95% CI 1.26–1.36), while the aPR for household or community exposures was 0.73 (95% CI 0.70–0.76), comparing the highest versus the lowest community incidence periods [9]. A prominent role of occupational exposure was also enlightened in the study by Carazo et al. They found that this was much more common than community and household exposures, although its percentage decreased significantly from the first to the third wave (from 91% to 40%), while non-occupational exposures showed the opposite trend (from 4% to 33%) [10]. However, several limitations affect the interpretation of the available data. In most studies, the source of infection was inferred from self-reported or retrospectively collected data, which led to recall bias. Furthermore, the high proportion of unknown exposures reflected the difficulties of contact tracing, especially during periods of high community transmission. These issues may lead to misclassification or underestimation of both occupational and extraprofessional exposures, thereby reducing the accuracy of assessing their relative contributions to the risk of infection among healthcare workers.

As a result, the uncertainty and inconclusiveness surrounding these results suggest that further investigation on this topic might be interesting. Furthermore, the lack of data on exposures during the final phases of the pandemic makes it even more difficult to achieve a comprehensive understanding of this issue, which is essential for the effective prevention and management of possible future pandemics.

The aim of this study was to investigate the dynamics of SARS-CoV-2 infection and the contributions of the professional and extra-professional exposures across all the pandemic phases among a large multi-centric Italian cohort of HCWs, in order to better understand the preventive measures to apply, to limit the spread of the infection not only among HCWs, but also among patients, as well as household and community contacts.

## 2. Materials and Methods

### 2.1. Study Design and Setting

The study included healthcare workers enrolled in the Horizon 2020 ORCHESTRA Project.

The study population comprised HCWs from the University Hospitals of Modena, Padua, Trieste, and Verona. Socio-demographic information and clinical data, including details on SARS-CoV-2 vaccination and prior infection, were collected retrospectively via online surveys. Data were collected until September 2022.

A complete description of the questionnaire’s structure and the details of the information collected were reported in a previous ORCHESTRA study [11].

This study followed the “Strengthening the Reporting of Observational Studies in Epidemiology” (STROBE) reporting guidelines and was approved by the Italian Medicine Agency (AIFA) and the Ethics Committee of the Italian National Institute of Infectious Diseases (INMI) Lazzaro Spallanzani (No. 436, 14 October 2021). The local ethical boards also approved the study.

### 2.2. Job Categories

For the analysis, the subjects were categorized into five primary occupational groups based on the tasks performed within healthcare institutions. The categories are delineated as follows:

- Administrative: Includes staff employed in management support, secretarial, accounting, document management, human resources, and other non-healthcare tasks.
- Nurses: Includes professional nurses and other care providers with nursing qualifications.
- Technicians: Includes personnel with technical-healthcare functions such as radiology technicians, laboratory technicians, prevention technicians, and the like. Also included are electromedical equipment technicians and other technical profiles supporting clinical practice.
- Physicians: Includes structured doctors, residents, and healthcare managers with clinical, surgical, or diagnostic functions.
- Others: Includes social and healthcare workers, auxiliary staff, and other professionals not included in the previous categories but still involved in healthcare.

Furthermore, to better address the actual occupational exposure, we also classified HCWs by the COVID patients care ward as follows:

- No-COVID wards: no direct contact with COVID patients during the occupational tasks.
- Low-intensity COVID wards: assistance to mild to moderate COVID patients.
- High-intensity COVID wards: assistance to severe COVID patients, for whom high-flow oxygen and invasive or non-invasive ventilation were performed.

These classifications were used to categorize the collected data and to support subsequent statistical analysis, highlighting any differences in infection rates among the professional groups considered.

### 2.3. Classification of Infection Periods

The infection period was classified based on the temporal correspondence between the chronological progression of SARS-CoV-2 viral variants and the phases of the vaccination campaign. To this end, four distinct epidemiological periods were defined:

- Not infected: subjects who did not contract a confirmed SARS-CoV-2 infection during the entire observation period.
- Pre-vaccination period (PVP): period prior to the start of the vaccination campaign (until 27 December 2020), characterized by the circulation of the first viral variants.
- Pre-Omicron period (POP): period following the start of the vaccination campaign but before the spread of the Omicron variant (from 28 December 2020 to 30 November 2021).
- Omicron variant period (OVP): period characterized by the predominance of the Omicron variant (starting from 1st December 2021 to 30 September 2022), with high transmissibility and reduced vaccine effectiveness in preventing symptomatic infection.

This temporal classification was used to stratify infected individuals according to the epidemiological and vaccination context in which the infection occurred, to assess the impact of variants and vaccine protection at different stages of the pandemic.

### 2.4. Statistical Analysis

Descriptive statistics were used to summarize demographic and occupational characteristics of the study population, stratified by job category (Administrative, Nurse, Technician, Physician, and Other), work unit risk, and period of acquired infection. Continuous variables were assessed for normality using the Shapiro–Wilk test. As most of them did not follow a normal distribution, they were presented as medians and I–III quartiles, while categorical variables were reported as absolute frequencies and percentages.

A further descriptive analysis was conducted among individuals who experienced one or more SARS-CoV-2 infections, and comparisons were made according to infection status (No infection, One infection, More than one infection). Additional analyses were performed among infected subjects according to the risk level of their working unit, which was classified into no COVID patient wards, Low-intensity COVID wards, and High-intensity COVID wards.

Associations between infection status and demographic or occupational characteristics were evaluated using the chi-square test (for categorical variables) or the Kruskal–Wallis test (for non-normally distributed continuous variables).

To investigate factors associated with SARS-CoV-2 infection during different pandemic periods (Not infected, Pre-vaccination, pre-Omicron variant, and Omicron variant), two multinomial logistic regression models were fitted. Variables were selected for inclusion in multinomial models when significant in univariable analysis. Moreover, we excluded variables with unbalanced distribution (country of birth and ethnicity) or collinear with other variables (age collinear with seniority) or assessed after the outcome (previous infection). The first model was applied to the entire cohort with complete information (n = 4581), the dependent variable was the infection period (0 = not infected as reference, 1 = PVP, 2 = POP, 3 = OVP), while explanatory variables included sex, comorbidity status, years of seniority (0–5, 6–15, >15 years), and work shifts (day or night). The second model was applied to infected individuals with complete information (n = 1648), the dependent variable was coded as 0 = PVP as reference, 1 = POP, 2 = OVP, and work unit risk (no, low, or high risk) and source of infection (extra-professional, occupational, and unknown) were added to the above-mentioned explanatory variables. Results were expressed as Relative Risk Ratios (RRR) with corresponding 95% confidence intervals (95% CI). The goodness-of-fit of the model was evaluated by the likelihood ratio test of the fitted model with respect to the intercept-only model and McFadden’s pseudo-R^2^. Additionally, sensitivity analyses were performed by excluding administrative staff and infected HCWs without information on infection source.

All statistical analyses were performed using STATA software, version 18.0 (StataCorp, College Station, TX, USA). A *p*-value < 0.05 was considered statistically significant.

## 3. Results

### 3.1. Descriptive Analysis

The study population (n = 5576) consisted mainly of nurses (34.7%) and other healthcare professionals (25.8%). Most participants were female (75.8%), although physicians had a relatively higher proportion of males (40.1%).

The median age was 47 years (I-II quartile: 33–55), with physicians being the youngest (35 years, 30–48) and administrative staff the oldest (54 years, 46–59).

Overall, 21.9% reported at least one comorbidity, more frequently among administrative staff (31.6%) and less among physicians (16.7%).

Most SARS-CoV-2 infections occurred during the OVP (69.7%), especially among physicians and nurses, whereas infections during the PVP were more common among nurses (Table 1).

As regarding occupational exposure, most participants (54.7%) worked only day shifts, particularly administrative staff (91.3%), while night shifts were more common among physicians (56.1%) and nurses (54.6%).

Overall, 43.5% had over 15 years of experience, mainly nurses (57.7%), whereas physicians had generally a shorter seniority, with 55.4% having less than 5 years.

Most subjects (62.5%) worked in low-intensity COVID wards; high-risk units were more frequent among nurses and physicians, while administrative staff mainly worked in non-COVID settings (82.1%).

Among those infected once, testing was mainly symptom-driven (41.5%), followed by contact tracing (26.8%) and routine screening (27.4%). Occupational exposure was reported in 26.8% of cases, especially among nurses, although most infections occurred outside the workplace (34.3%) or had an unknown source (38.9%).

Shared spaces were the most frequently identified setting for occupational exposure, while patient care was a relevant source for nurses and physicians (Table S1).

### 3.2. Analysis of the Infected Subjects

Of 5576 healthcare workers, 53.8% had no SARS-CoV-2 infection, 41.8% was infected once, and 4.4% had more than one infection (Table S2). The proportion of women slightly increased with the number of infections, but the association was not significant.

Mean age decreased significantly with increasing infections (No infection: 45.5 years; One infection: 43.6 years; More than one infection: 42.1 years; *p* < 0.001), indicating higher reinfection rates among younger workers.

Comorbidities did not differ significantly across groups.

Professional role was significantly associated with infection status (*p* < 0.001): nurses and physicians were more represented among infected workers, particularly nurses among those with multiple infections (45.1%). In contrast, administrative staff were more represented among uninfected workers (10.8%) compared to those with repeated infections (5.3%).

Seniority was also associated with infection (*p* = 0.001), with less experienced workers who are more frequently reinfected.

A strong association (*p* < 0.001) was also observed between infection status and the work units. Workers in high-intensity COVID units were more frequently represented among uninfected (65.1%) than among those who had multiple infections (62.5%; difference not large but consistent in trend). Conversely, no-COVID unit workers were more common among uninfected individuals (15.1%) than among those reinfected (5.7%).

Night-shift work was associated with a higher likelihood of infection (*p* < 0.001), suggesting a potential role of shift work in increasing infection risk (Table S2).

An analysis restricted to infected workers (Table S3) showed that, among 2516 subjects with available data, 10.3% (n = 258) worked in “no-COVID wards”, 25.8% (n = 650) in “low-intensity COVID wards”, and 63.9% (n = 1608) in “high-intensity COVID wards”.

Work unit risk was strongly associated with shift type (*p* < 0.001): day shifts predominated in no COVID wards (98.7%), while night shifts were more frequent in low-intensity COVID wards (70.0%).

Seniority also differed significantly (*p* < 0.001), with more experienced staff (>15 years) mainly in no-COVID (54.4%) and high-intensity COVID (50.4%) units, and less experienced staff (<5 years) in low-intensity units (36.7%).

Job category was highly associated with exposure level (*p* < 0.001): administrative staff were concentrated in no-COVID wards, nurses predominated in low- and high-intensity COVID wards, physicians were more represented in low-intensity COVID units, and technicians mainly worked in high-intensity COVID areas.

Reasons for testing varied by exposure (*p* = 0.003): symptoms were the main cause overall, particularly in no-COVID units, while periodic screening was more frequent in low- and high-intensity COVID wards. Reported exposure source also differed (*p* < 0.001): occupational exposure was more common in low-intensity COVID units, extra-work exposure was more common in no-COVID units, and uncertainty remained high across groups.

Meetings or shared spaces were the main setting in no-COVID units, whereas patient care activities predominated in low- and high-intensity COVID wards.

Finally, PPE use at the time of infection was significantly associated with exposure level (*p* = 0.009), with infections despite PPE more frequently reported in low- and high-intensity COVID units than in no-COVID settings.

### 3.3. Analysis of the Infection Trend by Pandemic Period

The infection trend throughout the pandemic, although significantly different, was rather similar in both men and women. Indeed, infections significantly decreased from the PVP to the POP in both genders, but rose again in the OVP reaching a cumulative incidence > 30%. Comorbidities were associated with a higher risk of infection before the vaccination campaign, but with a lower risk during the OVP. HCWs with longer seniority (>15 years), with respect to the other HCWs, experienced a higher incidence of infection in the first phase, but a lower incidence in the post-vaccination periods so that overall, they had a significantly lower percentage of infected subjects over the entire pandemic. Night shift workers, as compared to daily workers, presented a higher proportion of infected both over the entire observation period (52.4% vs. 43.8%), and during all the three stages of the pandemic (Table 2).

As regards infected HCWs, most infections (<72%) occurring in no COVID or low risk COVID wards took place in the OVP, while about one third of infections occurring in high risk COVID ward took place in the pre-vaccination period. The vast majority of infections acquired through extra-professional contacts (80.6%) occurred in the OVP, while those acquired through occupational exposure were more distributed over time, with 37% occurring before the vaccination campaign (Table 2 and Figure S1).

Regarding the testing purpose, the percentage of HCWs infected with SARS-CoV-2 but without symptoms was higher when the test was administered as part of periodic screening than after close contact with infected individuals (28.5% versus 16.4%; *p* < 0.001). These percentages were, respectively, 33.3% and 18.6% during the pre-vaccination period, peaked to 39.6% and 23.2% during the pre-Omicron period, and decreased to 25.4% and 14.8% in the Omicron-variant period. Looking at the total of asymptomatic infection detected by screening tests, more than 55% of them were identified during screening surveillance, ranging from 59.2% in the PVP to 54.9% during the OVP (Table S4).

### 3.4. Multivariable Analysis Results

Table 3 presents the results of two different multinomial logistic regression models: the effect of sex, comorbidity, seniority, and work shifts was evaluated on the entire sample with complete information taking as reference non-infected workers, while the effect of work unit risk and infection source was evaluated on infected individuals taking as reference the pre-vaccination period. Multivariable analyses substantially confirmed the results of univariable analyses (Table 2).

Night shift work was associated and increased risk of infection in all periods, with a stronger effect in the first two phases. Seniority was inversely related to the risk of infection in the last period only (RRR = 0.0.73, 95% CI 0.63–0.86). HCWs with comorbidities were at higher risk of infection during the PVP and female HCWs during the POP.

With respect to the pre-vaccination period, infections from occupational exposure were less likely than infections from extra-professional contacts in the POP (RRR = 0.39, 0.25–0.61) and OVP (RRR = 0.22, 0.16–0.29).

Results did not substantially change when excluding the administrative staff or infected individuals with unknown source of infection.

## 4. Discussion

Our findings, over the entire pandemic, including the different epidemiological phases and the surge of subsequent variants of concern, showed a very high cumulative incidence of SARS-CoV-2 infections among HCWs (46.2%). Of them, 70% occurred during the OVP, while the lowest incidence was during the post-vaccination period (10%). These results are in line with previous studies and seem to confirm, on one hand, the high effectiveness of vaccines against the first variants of concern and, on the other hand, the significantly greater transmissibility of the Omicron variant compared to previous ones [6,12,13,14,15,16].

Regarding the reason why the diagnostic test was performed, the onset of symptoms was the most frequent across all job categories (41.5%). On the other hand, periodic screening showed significant differences by job title. In particular, it was higher among nurses (32.6%) than in administrative staff (16.0%). This could be explained by differences in screening policies depending on whether direct patient care was provided (weekly, biweekly, or monthly). The high proportion of asymptomatic infections detected by periodic testing (55%) confirms that screening tests are effective in ensuring early detection of positives, especially when asymptomatic, and can prevent the spread of infection. In line with this hypothesis, Hellewell et al. reported that PCR screening can detect the majority of asymptomatic cases before symptom onset [17]. Moreover, in a previous study involving one of the Cohorts included in this analysis (University Hospital of Padua), the Authors reported that about a quarter of the total infections detected by screening tests were asymptomatic, highlighting the high incidence of asymptomatic infections among HCWs [18]. This allowed HCWs to leave the workplace promptly and avoid contact with patients and colleagues.

Contact with a positive accounted for a minority of cases (26.8%). Its frequency was higher among administrative staff (28.4%) than among nurses and physicians (27.3% and 25.3%), which may seem unexpected. However, it could be explained by the fact that this category included all contacts, not only those at work, and that the test was performed only when the exposure was classified as high risk (contacts without personal protective equipment). This kind of exposure was more frequent in extraprofessional settings than in hospital wards. Indeed, only 46% of employees in no COVID ward reported using PPE during contacts with infection sources, while staff working in COVID ward reported it in more than 60% of occasions, largely during patient assistance. In line with these results, Boomsma et al. found that exposures in which both HCWs and positive subjects were unmasked accounted for 20.3% of contacts in the community, compared with only 10% during patient care [19]. Regarding the source of infection, the percentage of unknown origin is the most frequent (38.9%). This finding is in agreement with previous studies, even if lower (Boomsma et al., 70%; Kwon et al., 67%; Shaw et al., 58%; Wight et al., 53%) [7,8,19,20]. This could be due to difficulty tracing contacts, especially during high-incidence periods, as well as the risk of infection from asymptomatic cases.

To better understand workplace exposure circumstances, we also collected data on the exposure occasion during work. The vast majority (59.7%) was undetected. However, significant differences were found among job titles. Assistance activity represented about a quarter of the occupational infection sources among physicians and one-sixth among nurses, and high-risk contacts with other HCWs presented a similar frequency. An even more relevant impact of exposures among HCWs was reported by Emecen et al. They investigated 329 clusters in health settings and found that almost 4 out of 5 occurred through HCW-to-HCW contact [21]. Similar results were described in the study by Farah et al. Among over 2200 occupational exposures, 57% of medium or high-risk contacts occurred between HCWs. Moreover, the risk of infection after exposure to a positive coworker had an OR of 3.22, as compared to patient contact [22]. This effect could be explained by HCWs perceiving contacts with colleagues as lower risk. As a result, they were less prone to use personal protective equipment and to maintain physical distancing in these contexts. However, it is crucial to point out that the prevalence of undetected exposure occasions significantly limits the reliability of these conclusions.

Multivariable analysis focused on the risk of infection during pandemic periods. Job experience did not affect the risk during PVP and POP, while during OVP a significant reduction in infection risk was found among HCWs with more than 15 years of job experience. These results are in line with those reported in previous ORCHESTRA studies. Indeed, in that research, we found a non-significant effect of age, which is strictly related to seniority, during POP, but a significant reduction in the last phase of the pandemic [6,13,23]. Therefore, these data suggest that job experience is not a major determinant of infection among HCWs.

We analyzed potential job exposure by classifying HCWs into three categories (no, low, or high risk). In multivariable analysis, we used these categories instead of the job title because they better reflect the actual occupational exposure, since HCWs with the same job title may be assigned to very different contexts, leading to possible misclassification of real exposure. The multinomial logistic regression detected a significant difference among work unit risk only during the OVP, with a significantly lower RRR for high-intensity COVID wards, as compared to no-risk wards. This could be related to the changes in the source of exposure throughout the different phases of the pandemic, limiting the relevance of occupational exposure in the latter phases of the pandemic.

Indeed, infections from occupational exposure were two-three times more common than those from extra-professional source in the PVP, while the pattern completely reversed in OVP As a result, occupational sources lost their key role over time. These results are in line with the findings of Billock et al. During the period between March 2020 and March 2021, they observed a higher prevalence of occupational exposures, with an adjusted prevalence ratio for reporting occupational contacts compared to community contacts of 1.31 (95% CI 1.26–1.36) [9]. Moreover, Cegolon et al., in a study involving one of the Cohorts included in this study, found that occupational exposure significantly decreased from 42.5% during the PVP to 15.6% after the Omicron variant surge [24]. Similarly, Carazo et al., in a study conducted between March 2020 and May 2021, found a marked prevalence of occupational exposure compared to non-occupational exposure in the early stages of the pandemic, with a gradual increase in the latter’s role in the following months (from 91% vs. 4% to 40% vs. 33%) [10]. They hypothesized that the increase in household transmission could be related to the loosening of restriction policies. Our assumption is that this effect may have been further strengthened in subsequent phases, due to personal protective equipment at work, a further lifting of preventive measures in community contexts, a lower perception of risk given the protective effect of vaccination, and the rapid increase of cases following the onset of the Omicron variant. On the other hand, Shaw et al. and Wight et al. concluded that, contrary to expectations, occupational exposure played only a marginal role among HCWs across all the pandemics [7,8]. Indeed, they found that this exposure accounted for less than 5% of the total. As in all studies, the results may have been affected by a high percentage of unattributable sources, leading to a significant underestimation of occupational cases. However, in agreement with our results, they showed that the greatest increase in cases related to non-occupational exposure occurred during periods of highest pandemic incidence, confirming that contact dynamics were strongly influenced by epidemiology and justifying the RRR trend highlighted in our study.

Our study has some limitations. First, exposure sources were self-reported and detected in only 60% of cases. This could have affected data reliability and limited comparability with other studies. On this point, it should be noted that most studies examining SARS-CoV-2 exposure among HCWs used self-reported data, as in our study. This reflects the widespread lack of an effective comprehensive tracking system throughout the pandemic, particularly during periods of high incidence, when the rapid spread of the infection made systematic contact tracing challenging.

Furthermore, the large proportion of exposures of unknown origin suggests that self-reporting alone may not be a sufficiently reliable method for contact tracing or for accurately estimating exposure risk across the various phases of the pandemic. In particular, although HWs underwent periodic testing, the large proportion of unknown sources showed that this was a crucial issue not only among the general population but also among HWs. This limitation introduced a bias that may have led to an underestimation or an overestimation of individual exposure settings, potentially reducing the effectiveness of infection prevention measures and procedures based on these data.

Additionally, data were collected retrospectively, potentially introducing recall bias. As a result, this may have led to misclassification of exposure source and increased the number of cases with unknown source, thereby reducing the overall reliability of the results. Finally, we classified them based on their activity with COVID patients; however, this reflected the prevalent activity throughout the study period, not necessarily the exclusive one. In fact, during different periods of the pandemic, HCWs underwent numerous reassignments, potentially leading to misclassification of actual occupational exposure.

This study also has some strengths. The multi-centric design of the study increased the generalizability of the results. Furthermore, most previous studies examined only short pandemic periods. Instead, ours covered the entire emergency phase of the pandemic, allowing us to analyze trends in exposure types over time. Lastly, we chose to classify HCWs by occupational exposure risk rather than by job title. We believe this classification better represents the actual exposure level than a professional role that does not account for whether activities were carried out in COVID wards or whether direct patient care was provided.

## 5. Conclusions

The higher risk of SARS-CoV-2 infection among HCWs was related not only to exposure to COVID-19 patients but also to contacts with positive coworkers, next of kin, and friends. Although preventive measures at work seemed effective, with the exception of the first phase of the pandemic, extra-professional exposures have played a decisive role throughout the pandemic, especially during periods of high incidence. As a consequence, in the event of new pandemics, it will be critical to implement procedures and guidelines to prevent infection outside the workplace, thereby better protecting the health of both HCWs and patients.

Standardized procedures to identify sources of infection beyond self-reporting, complementing self-reporting with structured epidemiological investigations, should be implemented. Such approaches would enable more reliable data collection on transmission dynamics and foster the development of prevention strategies better tailored to context-specific settings in potential future pandemic scenarios.

## Figures and Tables

**Table 1:** Baseline descriptive characteristics of the overall sample. Occupational exposure and COVID-19 infection.

	Total	Administrative	Nurse	Technician	Physicians	Other
	n = 5576	n = 531	n = 1935	n = 430	n = 1241	n = 1439
Sex						
Male	1315 (23.6%)	124 (23.4%)	264 (13.6%)	93 (21.6%)	498 (40.1%)	336 (23.3%)
Female	4227 (75.8%)	402 (75.7%)	1661 (85.8%)	333 (77.4%)	738 (59.5%)	1093 (76.0%)
Other/Not answered	34 (0.6%)	5 (0.9%)	10 (0.5%)	4 (0.9%)	5 (0.4%)	10 (0.7%)
Age *	47 (33–55)	54 (46–59)	49 (37–55)	48 (37–56)	35 (30–48)	46 (31–55)
Comorbidity						
Yes	1221 (21.9%)	168 (31.6%)	465 (24.0%)	99 (23.0%)	207 (16.7%)	282 (19.6%)
No	4352 (78.1%)	363 (68.4%)	1469 (76.0%)	331 (77.0%)	1033 (83.3%)	1156 (80.4%)
Not answered	3 (0.05%)	0 (0.0%)	1 (0.05%)	0 (0.0%)	1 (0.08%)	1 (0.07%)
Country of birth						
Italy	5325 (96.0%)	513 (96.8%)	1840 (95.8%)	419 (97.9%)	1194 (96.6%)	1359 (94.8%)
Other UE	108 (1.9%)	7 (1.3%)	45 (2.3%)	4 (0.9%)	18 (1.5%)	34 (2.4%)
Extra UE	109 (2.0%)	9 (1.7%)	34 (1.8%)	5 (1.2%)	24 (1.9%)	37 (2.6%)
Not answered	7 (0.1%)	1 (0.2%)	2 (0.1%)	0 (0.0%)	0 (0.0%)	4 (0.3%)
Ethnicity						
Caucasian	4410 (82.5%)	343 (70.6%)	1491 (80.0%)	367 (87.4%)	1196 (97.2%)	1013 (75.2%)
Other	546 (10.2%)	79 (16.3%)	220 (11.8%)	34 (8.1%)	30 (2.4%)	183 (13.6%)
Not specified	391 (7.3%)	64 (13.2%)	152 (8.2%)	19 (4.5%)	5 (0.4%)	151 (11.2%)
Previous infections						
None	2999 (53.8%)	324 (61.0%)	933 (48.2%)	243 (56.5%)	648 (52.2%)	851 (59.1%)
One	2331 (41.8%)	194 (36.5%)	891 (46.0%)	175 (40.7%)	541 (43.6%)	530 (36.8%)
More than one	246 (4.4%)	13 (2.4%)	111 (5.7%)	12 (2.8%)	52 (4.2%)	58 (4.0%)
Infection period						
Not infected	2999 (53.8%)	324 (61.2%)	933 (48.3%)	243 (56.5%)	648 (52.3%)	851 (59.2%)
Pre-vaccination period	528 (9.5%)	38 (7.2%)	237 (12.3%)	26 (6.0%)	102 (8.2%)	125 (8.7%)
Pre-Omicron variant	251 (4.5%)	25 (4.7%)	107 (5.5%)	18 (4.2%)	45 (3.6%)	56 (3.9%)
Omicron variant period	1791 (32.2%)	142 (26.8%)	656 (33.9%)	143 (33.3%)	445 (35.9%)	405 (28.2%)

* Median (p25–p75).

**Table 2:** Sociodemographic and occupational characteristics of the population by infection period.

	Not Infected	Period			*p* Value
		Pre-Vaccination	Pre-Omicron	Omicron Variant	
Gender					0.040
Men	734 (55.8)	126 (9,6)	42 (3.2)	413 (31.4)	
Women	2242 (53.1)	398 (9.4)	207 (4.9)	1373 (32.5)	
Comorbidity					0.028
No	2334 (53.7)	388 (8.9)	197 (4.5)	1428 (32.9)	
Yes	663 (54.4)	139 (11.4)	54 (4.4)	363 (29.8)	
Seniority					<0.001
0–5 years	699 (49.6)	120 (8.5)	65 (4.6)	525 (37.3)	
6–15 years	546 (52.9)	96 (9.3)	61 (5.9)	330 (32.0)	
>15 years	1376 (55.6)	258 (10.4)	98 (4.0)	742 (30.0)	
Work shifts					<0.001
Day	1710 (56.2)	248 (8.2)	125 (4.1)	959 (31.5)	
Night	1021 (47.6)	259 (12.1)	117 (5.5)	748 (34.9)	
Work unit risk *					<0.001
No COVID wards	-------	45 (15.7)	33 (11.5)	208 (72.7)	
Low-intensity COVID	-------	358 (19.4)	156 (8.5)	1331 (72.1)	
High-intensity COVID	-------	102 (32.3)	44 (13.9)	170 (53.8)	
Infection source					<0.001
Extra-professional	-------	97 (11.0)	74 (8.4)	712 (80.6)	
Occupational exposure	-------	254 (36.9)	73 (10.6)	361 (52.5)	
Unknown	-------	87 (19.9)	42 (9.6)	309 (70.6)	

* Referred to the period in which they got infected.

**Table 3:** Multinomial logistic regression analysis of factors associated with SARS-CoV-2 infection in different pandemic periods.

Reference			Analysis of All Subjects with Complete Information (*n* = 4581)		Analysis on Infected HCWs with Complete Information (*n* = 1648)	
			RRR (95% CI)	*p*-Value	RRR (95% CI)	*p*-Value
			Not-Infected		Pre-Vaccination Period	
Pre Vaccination Period	Sex	Male	1 reference		------	------
		Female	1.04 (0.82–1.33)	0.739	------	------
	Comorbidity	No	1 reference		------	------
		Yes	1.28 (1.01–1.63)	0.043	------	------
	Seniority	0–5 years	1 reference		------	------
		6–15 years	1.07 (0.80–1.45)	0.642	------	------
		>15 years	1.10 (0.85–1.41)	0.470	------	------
	Shifts	Day	1 reference		------	------
		Night	1.82 (1.48–2.24)	<0.001	------	------
Pre Omicron Variant Period	Sex	Male	1 reference		1 reference	
		Female	1.91 (1.29–2.83)	0.001	2.60 (1.46–4.64)	0.001
	Comorbidity	No	1 reference		1 reference	
		Yes	1.12 (0.80–1.59)	0.489	0.98 (0.62–1.55)	0.934
	Seniority	0–5 years	1 reference		1 reference	
		6–15 years	1.26 (0.86–1.85)	0.234	1.13 (0.68–1.92)	0.625
		>15 years	0.79 (0.56–1.12)	0.193	0.69 (0.44–1.10)	0.120
	Shifts	Day	1 reference		1 reference	
		Night	1.70 (1.27–2.27)	<0.001	1.08 (0.71–1.64)	0.709
	Work unit risk *°	No COVID wards	------	------	1 (reference)	
		Low-intensity COVID	------	------	0.73 (0.37–1.43)	0.359
		High-intensity COVID	------	------	0.88 (0.40–1.95)	0.759
	Infection * source	Extra professional	------	------	1 Reference	
		Occupational exposure	------	------	0.39 (0.25–0.61)	<0.001
		Unknown	------	------	0.71 (0.42–1.23)	0.225
Omicron Variant Period	Sex	Male	1 reference		1 reference	
		Female	1.11 (0.96–1.30)	0.165	0.90 (0.67–1.22)	0.510
	Comorbidity	No	1 reference		1 reference	
		Yes	1.00 (0.85–1.17)	0.956	0.78 (0.58–1.06)	0.115
	Seniority	0–5 years	1 reference		1 reference	
		6–15 yeas	0.83 (0.69–1.00)	0.054	0.83 (0.58–1.19)	0.317
		>15 years	0.73 (0.63–0.86)	<0.001	0.68 (0.50–0.91)	0.011
	Shifts	Day	1 reference		1 reference	
		Night	1.29 (1.13–1.48)	<0.001	0.96 (0.74–1.26)	0.785
	Work unit risk *°	No COVID wards	------	------	1 (reference)	
		Low-intensity COVID	------	------	0.86 (0.54–1.35)	0.511
		High-intensity COVID	------	------	0.50 (0.29–0.86)	0.013
	Infection source *	Extra professional	------	------	1 Reference	
		Occupational exposure	------	------	0.22 (0.16–0.29)	<0.001
		Unknown	------	------	0.49 (0.34–0.70)	<0.001

* Only in infected subjects. *° Referred to the period in which they got infected.

## Data Availability

The datasets generated during the current study are not publicly available because they contain sensitive data to be treated under data protection laws and regulations. Appropriate forms of data sharing can be arranged after reasonable request to the last authors.

## Supplementary Materials

The following supporting information can be downloaded at: https://www.mdpi.com/article/10.3390/healthcare14131872/s1, Table S1. Description of occupational exposure data. Table S2. Descriptive work exposure by infection status. Table S3. Description of occupational risk in infected subjects. Table S4. Testing purposing by symptoms and infection period. Figure S1. Percentage of infected individuals by work unit risk, across the three phases of the COVID-19 pandemic.

## Abbreviations

The following abbreviations are used in this manuscript:

AAR	Adjusted Attack Ratios
aPR	Adjusted Prevalence Ratio
HCWs	Healthcare Workers
AIFA	Italian Medicine Agency
INMI	Italian National Institute of Infectious Diseases
OVP	Omicron Variant Phase
POP	Pre-Omicron Period
PVP	Pre-Vaccination Period
RRR	Relative Risk Ratios
