# Supplementary material for: Impact of Occupational and Extra-Professional Exposure Across the Different Waves of the Pandemic on the Risk of SARS-CoV-2 Infection Among Healthcare Workers—The ORCHESTRA Project
Source: Healthcare (Basel). 2026 Jun 26;14(13):1872. doi: 10.3390/healthcare14131872 (PMC13361609; doi:10.3390/healthcare14131872)

Supplementary material

Table S1. Description of occupational exposure data

	Total (N=5,576)	Administrative (N=531)	Nurse (N=1,935)	Technician (N=430)	Physicians (N=1,241)	Other (N=1,439)
**Work shifts**						
Day shift	3,048 (54.7%)	485 (91.3%)	828 (42.8%)	280 (65.1%)	494 (39.8%)	961 (66.8%)
Night shift	2,146 (38.5%)	4 (0.8%)	1,056 (54.6%)	128 (29.8%)	696 (56.1%)	262 (18.2%)
Unknown	382 (6.8%)	42 (7.9%)	51 (2.6%)	22 (5.1%)	51 (4.1%)	216 (15.0%)
**Seniority***						
0-5 years	640 (27.5%)	44 (22.7%)	120 (13.5%)	32 (18.3%)	300 (55.4%)	144 (27.2%)
6-15 years	431 (18.5%)	47 (24.2%)	172 (19.3%)	35 (20.0%)	69 (12.8%)	108 (20.4%)
>15 years	1015 (43.5%)	84 (43.3%)	514 (57.7%)	95 (54.3%)	101 (18.7%)	221 (41.7%)
Unknown	245 (10.5%)	19 (9.8%)	85 (9.5%)	13 (7.4%)	71 (13.1%)	57 (10.7%)
**Work unit risk**						
No patient care	694 (12.4%)	436 (82.1%)	61 (3.2%)	20 (4.7%)	37 (2.9%)	140 (9.7%)
Low-intensity COVID wards	3483 (62.5%)	74 (13.9%)	1273 (65.8%)	381 (88.6%)	816 (65.8%)	939 (65.3%)
High-intensity COVID wards	1220 (21.9%)	8 (1.5%)	578 (29.8%)	23 (5.3%)	370 (29.8%)	241 (16.7%)
Unknown	179 (3.2%)	13 (2.5%)	23 (1.2%)	6 (1.4%)	18 (1.5%)	119 (8.3%)
**Testing purposing***						
Periodic screening	639 (27.4%)	31 (16.0%)	291 (32.6%)	41 (23.4%)	131 (24.2%)	145 (27.3%)
Contact with positive	625 (26.8%)	55 (28.4%)	243 (27.3%)	58 (33.2%)	137 (25.3%)	132 (24.9%)
Symptoms	966 (41.5%)	98 (50.5%)	326 (36.6%)	69 (39.4%)	251 (46.4%)	222 (41.9%)
Unknown	101 (4.3%)	10 (5.1%)	31 (3.5%)	7 (4.0%)	22 (4.1%)	31 (5.9%)
**Source of infection****						
Occupational exposure	690 (26.8%)	29 (14.0%)	335 (33.4%)	30 (16.0%)	153 (25.8%)	143 (24.3%)
Extra-professional exposure	885 (34.3%)	77 (37.2%)	313 (31.3%)	83 (44.4%)	216 (36.4%)	196 (33.3%)
Unknown	1002 (38.9%)	101 (48.8%)	354 (35.3%)	74 (39.6%)	224 (37.8%)	249 (42.4%)
**Occasion of occupational exposure**						
Meeting / Canteen	161 (23.3%)	25 (86.2%)	54 (16.1%)	15 (50.0%)	38 (24.8%)	29 (20.3%)
Assistance activity	117 (17.0%)	0 (0.0%)	57 (17.0%)	4 (13.3%)	34 (22.2%)	22 (15.4%)
Unknown	412 (59.7%)	4 (13.8%)	224 (66.9%)	11 (36.7%)	81 (53.0%)	92 (64.3%)

*Only in subjects infected once. ** Only in positive subjects

Table S2 Descriptive work exposure by infection status

	**Total** (n=5,576)	**No infection** (n=2,999)	**Infected once** (n=2,331)	**More than one infection** (n=246)	**p-value**
**Sex**					0.143
Male	1,315 (23.6%)	734 (24.5%)	530 (22.7%)	51 (20.7%)	
Female	4,227 (75.8%)	2,242 (74.8%)	1,792 (76.9%)	193 (78.5%)	
Other/Not answered	34 (0.6%)	23 (0.8%)	9 (0.4%)	2 (0.8%)	
**Age***	47 (35-55)	49 (34-56)	45 (33-54)	42 (30-52)	<0.001
**Comorbidity**#					0.917
Yes	1,221 (21.9%)	663 (22.1%)	505 (21.7%)	53 (21.5%)	
No	4,352 (78.1%)	2,334 (77.9%)	1,825 (78.3%)	193 (78.5%)	
**Job**					
Administrative	531 (9.5%)	324 (10.8%)	194 (8.3%)	13 (5.3%)	<0.001
Nurse	1,935 (34.7%)	933 (31.1%)	891 (38.2%)	111 (45.1%)	
Technician	430 (7.7%)	243 (8.1%)	175 (7.5%)	12 (4.9%)	
Physicians	1,241 (22.3%)	648 (21.6%)	541 (23.2%)	52 (21.1%)	
Other	1,439 (25.8%)	851 (28.4%)	530 (22.7%)	58 (23.6%)	
**Seniority** #					
0-5 years	1,410 (28.6%)	699 (26.7%)	640 (30.7%)	71 (32.9%)	0.001
6-15 years	1,033 (21.0%)	546 (20.8%)	431 (20.7%)	56 (25.9%)	
>15 years	2,480 (50.4%)	1,376 (52.5%)	1,015 (48.7%)	89 (41.2%)	
**Work unit risk** #					
No COVID patient wards	694 (12.8%)	436 (15.1%)	244 (10.7%)	14 (5.7%)	<0.001
Low-intensity COVID wards	1,220 (22.6%)	570 (19.8%)	572 (25.2%)	78 (31.8%)	
High-intensity COVID wards	3,483 (64.5%)	1,875 (65.1%)	1,455 (64.1%)	153 (62.5%)	
**Work shifts**#					
Day shift	3,048 (58.7%)	1,710 (62.6%)	1,213 (54.6%)	125 (51.4%)	<0.001
Night shift	2,146 (41.3%)	1,021 (37.4%)	1,007 (45.4%)	118 (48.6%)	

*median, I-III quartile

#There was unknown information for: comorbidity (n=3), years of working experience (n=653), work unit risk (n=249), and work shifts (n=382)

Table S3. Description of occupational risk in infected subjects

	Total n=(2,516)	No COVID wards n=(258)	Low-intensity COVID wards n=(650)	High-intensity COVID wards n=(1,608)	p-value
**Work shifts**[#]					
Day shift	1,313 (54.3%)	234 (98.7%)	192 (30.0%)	887 (57.4%)	<0.001
Night shift	1,107 (45.7%)	3 (1.3%)	447 (70.0%)	657 (42.6%)	
**Seniority**[#]					
0-5 years	688 (30.5%)	51 (21.5%)	212 (36.7%)	425 (29.5%)	<0.001
6-15 years	473 (21.0%)	57 (24.1%)	126 (21.8%)	290 (20.1%)	
>15 years	1,095 (48.5%)	129 (54.4%)	240 (41.5%)	726 (50.4%)	
**Job**					
Administrative	202 (8.0%)	166 (64.3%)	3 (0.5%)	33 (2.1%)	<0.001
Nurse	993 (39.5%)	27 (10.5%)	317 (48.8%)	649 (40.4%)	
Technician	184 (7.3%)	3 (1.2%)	11 (1.7%)	170 (10.6%)	
Physicians	589 (23.4%)	16 (6.2%)	193 (29.7%)	380 (23.6%)	
Other	548 (21.8%)	46 (17.8%)	126 (19.4%)	376 (23.4%)	
**Testing purposing**					
Periodic screening	700 (27.8%)	46 (17.8%)	208 (32.0%)	446 (27.7%)	0.003
Contact with positive	686 (27.3%)	78 (30.2%)	159 (24.5%)	449 (27.9%)	
Symptoms	1,025 (40.7%)	123 (47.7%)	258 (39.7%)	644 (40.0%)	
Other	105 (4.2%)	11 (4.3%)	25 (3.8%)	69 (4.3%)	
**Source of infection**					
Occupational exposure	682 (27.1%)	37 (14.3%)	233 (35.8%)	412 (25.6%)	
Extra working exposure	862 (34.3%)	105 (40.7%)	200 (30.8%)	557 (34.6%)	<0.001
I don't know	972 (38.6%)	116 (45.0%)	217 (33.4%)	639 (39.7%)	
**Occasion of occupational exposure**					
Meeting / Canteen	159 (23.3%)	31 (83.8%)	94 (22.8%)	34 (14.6%)	<0.001
Assistance activity	116 (17.0%)	0 (0.0%)	77 (18.7%)	39 (16.7%)	
I don't know	407 (59.7%)	6 (16.2%)	241 (58.5%)	160 (68.7%)	
**Use of PPE**[##]					
Yes	447 (65.5%)	17 (46.0%)	286 (69.4%)	144 (61.8%)	0.009
No	95 (14.0%)	8 (21.6%)	57 (13.8%)	30 (12.9%)	
I don't know	140 (20.5%)	12 (32.4%)	69 (16.8%)	59 (25.3%)	

[#]There were unknown information for work shift (n=96), years of experience (n=260). [##] PPE=Personal Protective Equipment

Table S4. Testing purposing by symptoms and infection period

Pandemic period	Testing purposing	Yes (%)	No (%)
Pre-vaccination period	Periodic screening	84 (66.7)	42 (33.3)
	Contact with positive	101 (81.5)	23 (18.6)
	Unknown	5 (62.5)	3 (37.5)
Pre-Omicron Period	Periodic screening	32 (60.4)	21 (39.6)
	Contact with positive	43 (76.8)	13 (23.2)
	Unknown	4 (100)	0
Omicron variant period	Periodic screening	296 (74.6)	101 (25.4)
	Contact with positive	340 (85.2)	59 (14.8)
	Unknown	27 (77.1)	8 (22.9)

Figure S1. Percentage of infected individuals by work unit risk, across the three phases of the COVID-19 pandemic.

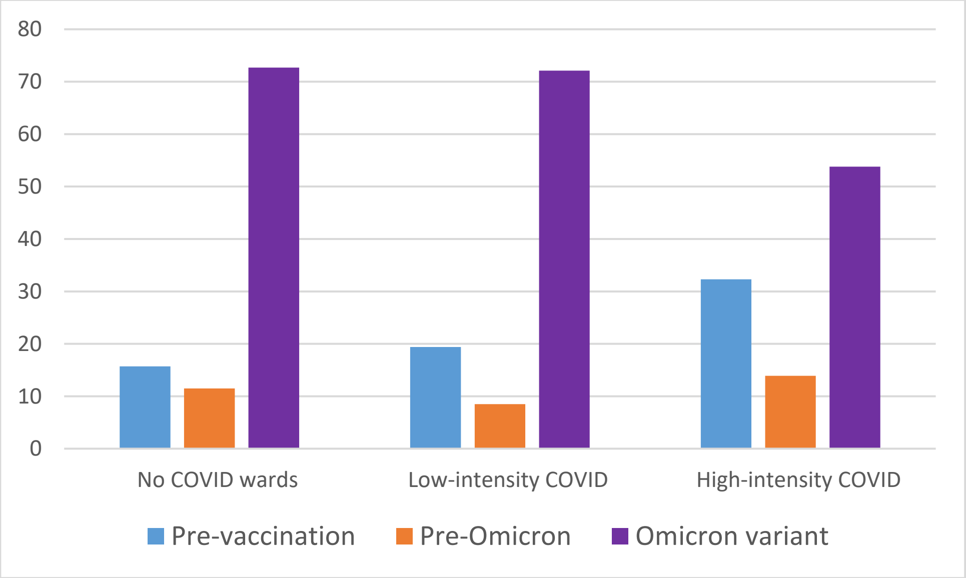

Supplement: Supplementary file 1 [file healthcare-14-01872-s001.zip › healthcare-4289479-supplementary.pdf]
